# Sexually transmitted infections on the border between Suriname and French Guiana: A scoping review

**DOI:** 10.3389/fmed.2022.994964

**Published:** 2022-10-05

**Authors:** Mathieu Nacher, Aylosius Koendjbiharie, Céline Michaud, Sébastien Rabier, Cyril Leborgne, Cyril Rousseau, Aude Lucarelli, Camille Thorey, Adriana Gonzales, Fredrik Terlutter, Nadia Thomas, Benoit Van Gastel, Sophie Biacabe, Marja Van Eer, Stephen Vreden, Najeh Hcini, Lycke Woittiez

**Affiliations:** ^1^INSERM CIC1424 Centre d’Investigation Clinique Antilles Guyane, Cayenne, French Guiana; ^2^Regional Health Services (RGD), Moengo, Suriname; ^3^Centre Hospitalier Andrée Rosemon, Cayenne, French Guiana; ^4^Centres Délocalisés de Prévention et de Soins, Cayenne, French Guiana; ^5^Centre Hospitalier de Cayenne, Cayenne, French Guiana; ^6^Centre Hospitalier de l’Ouest Guyanais, Saint-Laurent-du-Maroni, French Guiana; ^7^Croix Rouge Française, Cayenne, French Guiana; ^8^Agence Régionale de Santé Guyane, Cayenne, French Guiana; ^9^Diakonessen Hospital, Paramaribo, Suriname; ^10^Academisch Ziekenhuis Paramaribo, Paramaribo, Suriname

**Keywords:** border, Suriname, French Guiana, sexually transmitted infections, HIV, congenital syphilis, testing

## Abstract

**Purpose:**

The Maroni basin –delineating the border between Suriname and French Guiana— presents sociocultural, geographical and economic circumstances that have been conducive to the circulation of sexually transmitted infections and to delays in diagnosis and care. Given the scarcity of published data, we aimed to describe different sexually transmitted infections along the Maroni and to gain a broader understanding of the epidemiologic situation.

**Methods:**

We conducted a scoping review of the efforts to approach the problem of sexually transmitted infections in this complex border area. Temporal trends were plotted and crude numbers were divided by local population numbers.

**Results:**

For HIV, despite increasing testing efforts, most patients still present at the advanced HIV stage (median CD4 count at diagnosis is < 20 per mm^3^), and 25% of patients in Saint Laurent du Maroni were lost to follow-up within 6 years. However, progress on both sides has led to a decline in AIDS cases and mortality. Despite a rapid increase in the 1990’s along the Maroni, the current HIV prevalence seemed lower (0.52%) in the rural villages than in coastal urban centers (> 1%). High risk HPV infection prevalence among women reaches 23.3%. The incidence of gonorrhea was 4.2 per 1,000 population aged 15-59. For chlamydiasis it was 3.4 per 1,000 population aged 15-59. For syphilis, the incidence was 2.5 per 1,000 population aged 15-59. Gonorrhea, chlamydiasis, hepatitis B detection increased over time with greater testing efforts and new diagnostic tests. Since the COVID-19 epidemic, congenital syphilis has dramatically increased in Saint Laurent du Maroni reaching 808 per 100,000 live births.

**Conclusion:**

Sexually transmitted infections seemed more prevalent in Saint Laurent du Maroni –the sole urban center—than in the remote villages along the Maroni. The syndromic approach and the heterogeneity of diagnostic platforms presumably overlook most infections in the region. Therefore, a concerted approach and a shared diagnostic upgrade with molecular diagnosis and rapid diagnostic tests seem necessary to reduce the burden of sexually transmitted infections on both sides of the Maroni. Congenital syphilis resulting from COVID-19 disruption of health services requires urgent attention.

## Introduction

Border areas often lead to complex social and economic exchanges. Borders may also constrain human movements and diseases. Transmission, prevention and care of HIV and other sexually transmitted diseases are also influenced by borders (1–5). Social inequalities may foster transactional sex and sex work, and more generally, having casual sex partners on the other side of the border may be a way to escape social scrutiny. Differences in health care access or stigma may also influence some patients to seek care on the other side of the border.

The Guiana shield is a region where the prevalence of HIV has exceeded 1% for over 3 decades. The per capita health expenditure in 2018 was 474 US dollars in Suriname vs. 2,500 US dollars in French Guiana (6, 7). The border between Suriname and French Guiana is 520 km long, and mostly unenforced, apart from the post at Albina (5,247 inhabitants) and Saint Laurent du Maroni (45,000 inhabitants). The rest of the border is mostly surrounded by Amazonian forest where villages can only be reached by canoe or small aircrafts. The population of Eastern Suriname is around 31,000 persons (Marowijne 18,294 and Sipaliwini’s Tapanahoni sub District 13,808) and that of western French Guiana is circa 90,000 persons (it quadrupled since the 1990s). The population is mostly composed of Maroon tribes, and to a lesser extent, Amerindian tribes who all live on both sides of the Maroni river. When relatives and friends are scattered on both sides, the administrative dimension of the river is hence often virtual. This leads to what is called pendular migration, with people moving back and forth between sides (8). Given these continuous fluxes, it is key to act on both sides of the border and to coordinate the health professionals and Non-Government Organizations (NGOs) on both sides of the border. Each have national plans to implement, central authorities to report to, which leads to 2 distinct and poorly connected networks driven by their own internal constraints. However, in practice, the health professionals are dealing with the same diseases in the same populations; it is thus crucial to coordinate prevention and care on both sides of the border. In these remote areas epidemiologic surveillance and investigation are key to prioritize public health action for 2 main reasons: because of the often specific health problems in areas that are still undergoing the epidemiologic transition; and also the representations of health and disease along the Maroni may be quite different from coastal areas. Hence, data and expertise-sharing to shed light on both sides of the border are also crucial. Our objective here was to assemble researchers, clinicians and public health practitioners from both Suriname and French Guiana and to conduct a scoping review of the efforts to approach the problem of sexually transmitted infections in this complex border area. The approach will tackle drivers of sexual transmission and detail knowledge on different sexually transmitted infections for which there are published studies or available data, and search for temporal trends.

## The health structures along the Maroni

**FIGURE 1:**
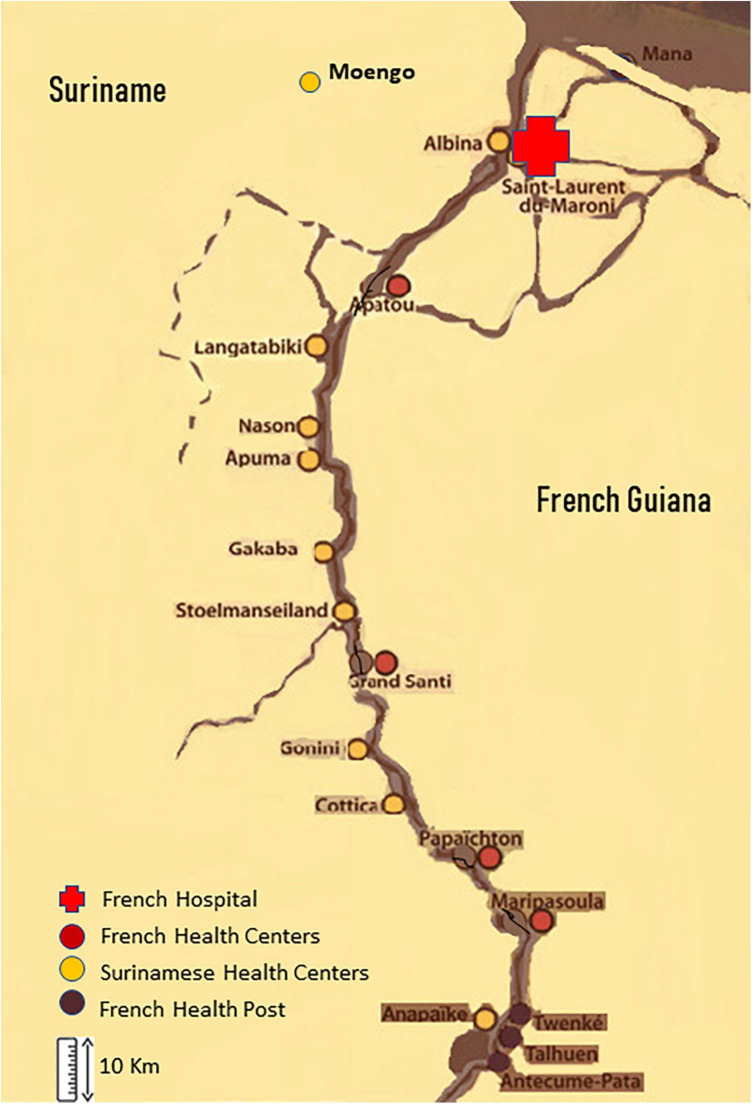
Health structures along the border between Suriname and French Guiana.

On the Surinamese side, there are primary care centers managed by the Regionale Gezondheidsdienst Suriname (RGD) in Moengo and Albina for the Marowijne. These centers provide preventive services, voluntary counseling and testing (VCT), laboratory, pharmacy and ambulance services, and treatment and care including home visits, data collection and dissemination for an evidence-based adaptation of public policy. Further down the Maroni river, in the Tapanahoni sub district, the health centers of the medical mission Medische Zending (MZ)) also provide primary care, including prevention, VCT, and treatment. The only tertiary referral structure is in Paramaribo, often hours away. In French Guiana, Saint Laurent du Maroni has a general hospital with an advanced diagnostic platform, Magnetic Resonance Imaging and Computerized Tomography scan, dialysis, Intensive Care Unit beds. There are private practitioners, laboratories and pharmacies. The health centers along the Maroni are administratively attached to Cayenne general hospital, the reference hospital. They are medicalized primary care structures that offer free health care to all and regular specialist missions (infectious diseases consultant, pediatrics, dermatologist); treatments are free and delivered through the central pharmacy in Cayenne hospital. The health posts on the upper Maroni are not medicalized but regular physician missions are provided. The different structures are mapped in Figure 1.

One of the very first steps of care is to get the patient some form of health insurance to cover the cost depending on the patients administrative and financial situation. Even undocumented migrants may access Aide Medicale Etat, or Soins Urgent, which allows to cover costs. This means that a sick patient crossing the Maroni from Suriname will be treated and, if necessary, hospitalized.

## HIV

Over half of the HIV 1 viruses circulating in French Guiana and Suriname are B_*CAR*_ lineages (9). Bayesian phylogeographic analyses identify different B_*CAR*_ lineages that arose after dissemination of a single variant from Trinidad and Tobago into French Guiana in the middle of the 1970s, subsequently spreading into Suriname, Brazil, and Guyana. The other French Guianese/Surinamese B_*CAR*_ lineages are thought to have come from Hispaniola into Suriname and French Guiana between the late 1970s and the mid-1980s, with multiple viral exchanges between countries. B_*PAN*_ lineages resulted from concurrent dissemination of B_*PANDEMIC*_ strains from North America into French Guiana and Suriname in the mid-1980s. There again, the virus crosses borders, reemphasizing the need for a regional approach. The HIV epidemic has hence been in the region for a long time, progressing at different speeds in different sexual networks (10).

In French Guiana, which has a population of 290,000, modeling using the SPECTRUM (AIM/CSAVER modules) and ECDC modeling tool suggested there are 3,600 HIV patients, 10% of whom were undiagnosed (11). A recent study adding health insurance data shows there were 3,047 patients on antiretroviral treatment in 2020.

As in the rest of French Guiana, transmission in Saint Laurent du Maroni is essentially heterosexual (87.7%); the unknown mode of transmission was next (8.7%), and mother to child transmission was 1.9%. Overall, 36% of patients are men. In Saint Laurent du Maroni (population 43,600), 594 persons were on antiretroviral drugs in 2020, a number increasing by 6.2% per year between 2016 and 2020. This represents a proportion of treated HIV patients in the population of 1.37%, perhaps a low estimate of the actual seroprevalence rate. The proportion of patients only followed by private practitioners increases regularly (24.4% in 2020); This gradual shift toward private practice was also observed for the most precarious patients who rapidly obtain insurance, which allows them to consult private practitioners. At Saint Laurent du Maroni hospital, 198(47.8%) of 414 outpatients were Surinamese. There are great testing efforts with diverse free options for persons to choose from: prescribed ELISA test in a laboratory in Saint Laurent, testing at health centers, at the hospital, STI clinics at the hospital or the red cross, mobile testing by NGOs and self-testing. Despite testing efforts, the cascade of care in Saint Laurent has remained under par (12).

**FIGURE 2:**
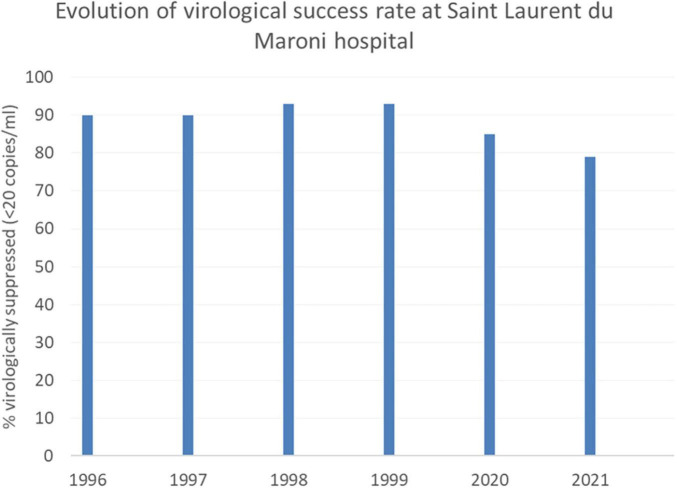
In 2020 and 2021, in Saint Laurent du Maroni HIV outpatient clinic, the proportion of virologically supressed patients dropped following COVID-19-related disruption of health services and border closure.

Although French recommendations promote systematic antiretroviral treatment for all since 2015, in the Saint Laurent du Maroni hospital cohort in 2022, only 75.4% of patients were on antiretroviral treatment, 16.4% had stopped and 8.2% were treatment naïve. The most frequent regimens were the following: Dolutegravir/abacavir/lamivudine (Triumeq©(26%); Elvitegravir, emtricitabine, tenofovir alafenamide, cobicistat (Genvoya©) (18%); Ritonavir + Atazanavir + Emtricitabine/Tenofovir (10.50%); Ritonavir + Darunavir + Emtricitabine/Tenofovir (8.29%); and Bictegravir, emtricitabine, tenofovir alafenamide (Biktarvy©)(7,73%). Figure 2 shows the evolution of virological success in the past 5 years with a substantial decline from 93 to 79% since the beginning COVID epidemic. Among Surinamese patients, virological success was only achieved for 70%, perhaps reflecting additional challenges to continue treatment due to border closure.

**FIGURE 3:**
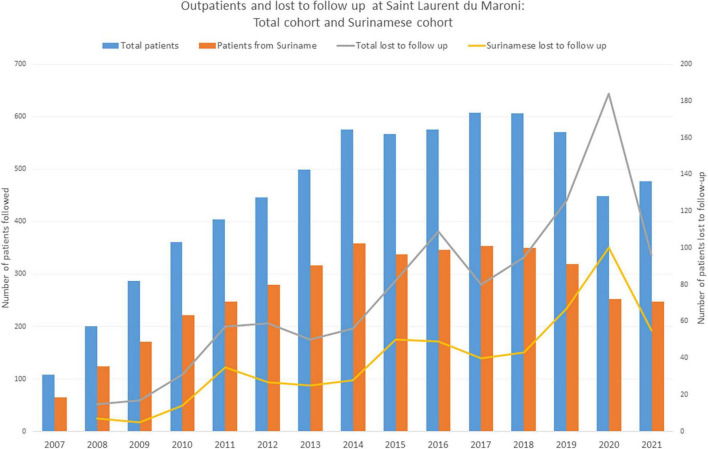
Persons from Suriname represent the majority of patients from Saint Laurent du Maroni’s HIV outpatient clinic. A substantial proportion of all patients are lost to follow-up, notably patients from Suriname. In 2020 and 2021, following disruption of health services and border closure, the number of outpatients seen for HIV follow-up declined significantly due to the impact of the COVID-19 epidemic.

There have so far been 416 patients lost to follow-up in the Saint Laurent hospital cohort (over 50 per year), 212 of whom were Surinamese. Figure 3 shows the temporal evolution of the proportion of patients lost to follow-up. It is estimated that 25% of HIV patients in Saint Laurent du Maroni are lost to follow-up within 6 years and that 65% of them are aged less than 40 years. Lost to follow-up patients were also usually successfully treated (80% had undetectable viral load), 41% had CD4 counts above 500 and 23.6% had CD4 counts below 200 per mm^3^. The sex ratio among the lost to follow-up was balanced.

**FIGURE 4:**
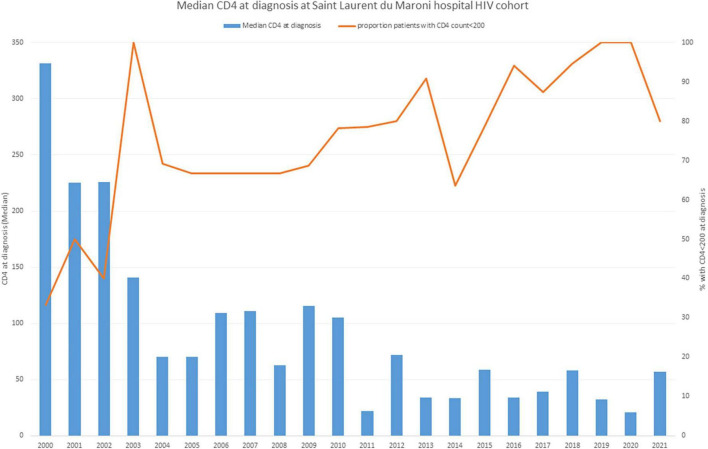
The median CD4 count at the time of diagnosis in Saint Laurent du Maroni tends to decline over time, with over 70% of new diagnoses with advanced HIV-disease.

**FIGURE 5:**
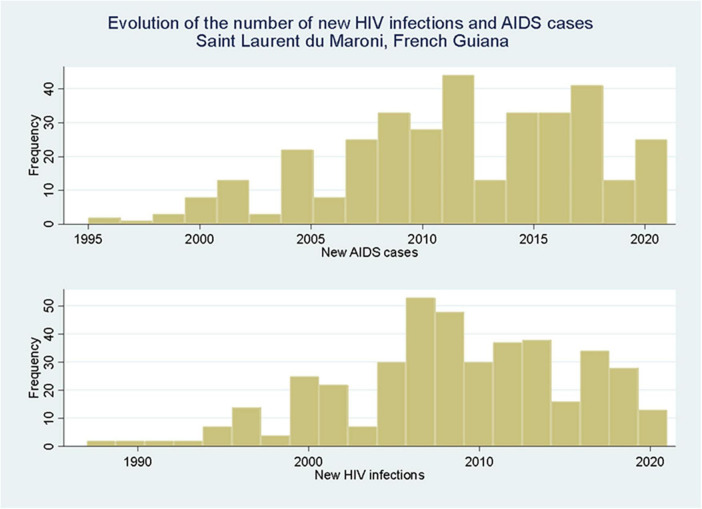
New HIV infections and new AIDS cases in Saint Laurent du Maroni show a peak around 2010 with a slow decline thereafter.

Figure 4 shows the very low CD4 count at diagnosis in Saint Laurent (over two thirds) with a significant linear trend indicating greater immunosuppression (*P* = 0.00002). Despite this, the number of new HIV infections and AIDS cases each year has gradually declined (Figure 5). It is of note that, in Saint Laurent du Maroni hospital, the main opportunistic infection is progressive disseminated histoplasmosis with 42% of hospitalized febrile HIV patients with less than 200 CD4 per mm^3^ being diagnosed with it and 85% of those with less than 50 CD4 per mm^3^ (13).

**FIGURE 6:**
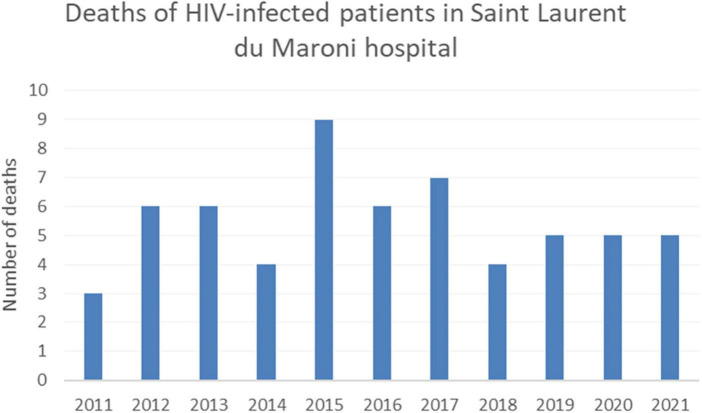
Evolution of the number of HIV-related deaths in Saint Laurent du Maroni.

Figure 6 shows that the number of deaths seems to have peaked in the middle of the decade, with perhaps a slight decline, which, however, needs confirmation with additional data. Overall, about 1% of the number of patients in Saint Laurent die each year. This number is substantially greater (about 6 fold) than the number of deaths in France divided by the number of PLWHIV (14, 15) and is presumably explained by the very late initial presentation of patients in Saint Laurent and the frequent follow-up interruptions.

On the Maroni river, data from the French health centers shows that the prevalence of HIV among pregnant women was zero in the early 1990’s but that within a decade it exceeded 1% (16). Overall, at the Saint Laurent maternity –which attracts women from the Maroni health centers and from Suriname— between 30 and 35 women with HIV give birth each year, a figure slightly above 1% of deliveries that has remained stable over time.

**FIGURE 7:**
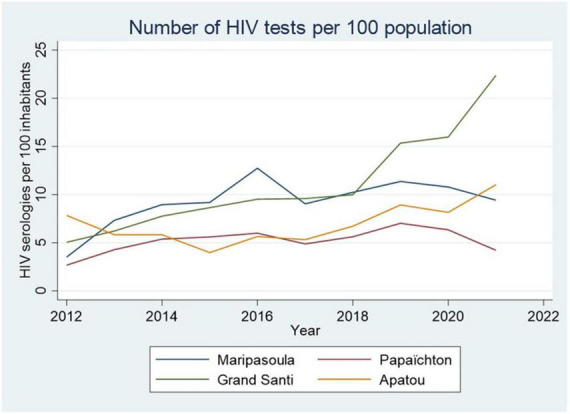
The evolution of the number of HIV tests in the health centers on the French side of the Maroni river shows a general increase over time. The disruptions due to COVID-19 have however had an impact in Maripasoula and Papaïchton.

In 2020, in the health centers on the French side of the Maroni river, there were 145 consulting HIV patients, mostly between Maripasoula and Apatou, 34.8% of whom were Surinamese. It is of note that in 2021, in Grand Santi HIV testing has recently increased and there were a total of 54 patients (0.6% of the total population), with 9 new patients all from Suriname. For patients in Maripasoula and Papaichton, the Takari project by the NGO AIDES provides health mediators and assistance to access health insurance for persons with HIV. The global virological suppression rate in the health centers was only 75%. Overall, since 2018 there are 214 known patients in the French health centers on the Maroni river servicing a population of approximately 41 000, which represents a prevalence of 0.52% (95% CI = 0.45-0.59%) whereas the overall prevalence in French Guiana is estimated to be between 1.18 and 1.35%. This low prevalence is consistent with a recent seroprevalence survey along the Maroni river that found an HIV prevalence of 0.47 on a sample of 666 persons. HIV testing activity in the health centers on the French side of the Maroni was at an average of 8.1 tests per 100 population; testing seemed on the increase (Figure 7), on par with most French regions; However, the levels of testing were less than half of recent estimates for French Guiana–20.3 tests per 100 inhabitants. Such data quantifying testing efforts on the Surinamese side were not available.

When looking at the proportion of positive HIV tests in the health centers, the average was 1.5% of positive tests, a positivity rate that is much higher than mainland France where it ranged from 0.07 to 0.33% and the overall proportion of positive tests in laboratories of French Guiana –0.69%. There is an apparent discrepancy between prevalence estimates and percentage of positive serologies at the health centers. This is not, however, a measure of true prevalence since patients may be tested more than once, notably in a context of frequent personnel turnover and patient identification difficulties.

Overall, these data suggest that the bulk of HIV infections gravitate around the city of Saint Laurent and that villages along the Maroni tend to have a lower prevalence—a finding that goes against common preconceptions in the region.

In Suriname, which has a population of 586,000, it is estimated that 5,200 persons are living with HIV (17). The annual number of new infections has declined since 2006, stabilizing around 500 new cases per year. In eastern Suriname, for Marowijne (18,000 population) there were only 45 known HIV patients followed at Moengo RGD primary health center. In the Tapanahoni subdistrict medical mission health centers along the Maroni, there were only 5 HIV patients 2 of whom were followed in a health center in French Guiana. This is far from the country prevalence above 1% and suggests 2 mutually compatible hypotheses to explain this contrast: first, primary care centers may only see and treat a fraction of the HIV patients, the others –perhaps for fear of stigma in small towns—are either not diagnosed or treated, or seen in hospitals in the capital Paramaribo (about 2 h away) or in Saint Laurent du Maroni (across the river); the other hypothesis is that, in this rural area, the true prevalence is lower that in urban Paramaribo. This gap is a major interrogation for the Surinamese health system. The large number of Surinamese patients followed in Saint Laurent hospital suggests that a large fraction of the gap is explained by transborder migration. However, anonymized data does not allow to know for sure whether this hypothesis is correct. Nevertheless, this emphasizes the need for close collaboration between doctors on both sides so that they can consult their colleagues across the river and be aware of the patients’ treatment regimens thus avoiding adverse effects due to drug interactions.

During the past decade, there have been remarkable changes in antiretroviral treatment policy: between 2007 and 2012, it was estimated that 28.16% of patients never entered into care and only 25% of this cohort was virologically suppressed (< 1,000 copies/ml) (18). This meant that a large proportion of the HIV population was not virologically suppressed and thus remained infectious. The decision to change the treatment initiation at 350 CD4 in 2015 and 500 CD4 in 2017 has largely corrected this (18, 19). However, the problem of follow-up interruption remained pressing. At the Moengo primary health center, in Eastern Suriname, there used to be 45% lost to follow up patients and only 25% of those on treatment actually had viral load measurements (they needed a 2-h drive to the capital Paramaribo to get it done). The health center attempted a classical “buddy” system where a friend or relative accompanied the patient to enhance adherence and follow-up but this failed to impact the problem, presumably because of the fear of stigma in small populations (20). They then used nurses as “buddies” and, since 2016, this had a dramatic impact on follow-up with patients lost to follow-up dropping from 42% to less than 5%. The clinic also proposed to draw blood on site and send the tubes instead of the patient, which also led to all patients benefiting from viral load measurements. Overall, from 12.5% virological suppression in Eastern Suriname, the proportion of virologically suppressed is now 100% of those treated. This has presumably had a strong impact on transmission and on the incidence of AIDS and probably explains the gradual reduction of new HIV and AIDS cases witnessed at Saint Laurent du Maroni hospital (Figure 4). HIV treatment, viral load and CD4 measurements are free in Suriname. However, the patients have to pay for the visit to the doctor and the rest of the laboratory investigations.

The first line antiretroviral treatment strategies in Suriname today consist of Atripla (emtricitabine + tenofovir + efavirenz) or lamivudine + zidovudine + nevirapine (Duovir N) or, more recently, Tenofovir + Lamuvidine + Dolutegravir (TLD). Although, recent drug resistance studies on both sides of the border are lacking, studies from Suriname and French Guiana were reassuring (21, 22) and the gradual improvements of virological suppression rates do not suggest particularly high levels of resistance in a context of migration and follow-up interruptions.

**FIGURE 8:**
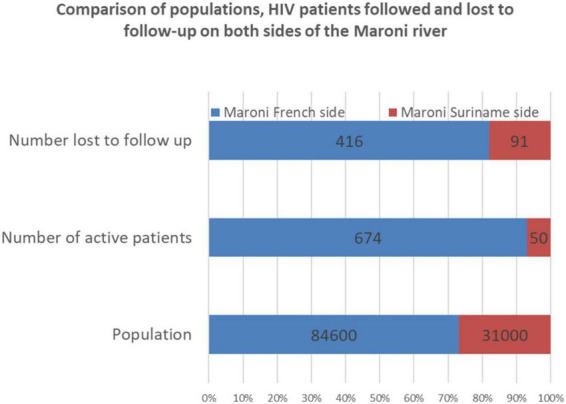
The blue and red bars show the population numbers **(bottom)**, patients **(middle)**, and lost to follow-up patients in French Guiana and Suriname. There is a greater demographic weight of the French side of the Maroni river but an even greater imbalence in HIV patients followed on the French side.

Figure 8 synthesizes the available data and shows the bulk of HIV seems to be on the French side of the Maroni, notably in the only city along the Maroni. Overall, a view embracing cross border aspects suggests that testing and treatment have led to improvements that should continue to reduce transmission and restore immunity. The problem of retaining patients in care remains acute: populations on the Maroni are often mobile when compared to urban populations; it seems that patients from small villages are frequently tempted –presumably to avoid stigma—to seek care in larger cities, Saint Laurent du Maroni being often closer than Paramaribo, this may be a simple explanation for why patients cross over; expected social benefits may also be a factor motivating some patients to seek care in Saint Laurent; in Saint Laurent, the COVID 19 epidemic (23) has apparently seriously undermined patient care with a substantial number not on treatment and a decline in virological success suggesting treatment interruptions due to lock downs, canceling of consultations, or, for some, fear of consulting the hospital. This underlines the fragility of past achievements; however, after the epidemic it seems likely that treatment coverage will improve again once the barriers to care are removed. Cross border efforts to monitor the evolution of the main indicators will be required. The present initiative of a shared epidemiological assessment should also facilitate patient-centered doctor to doctor collaboration. For instance, brazilians with HIV (often garimpeiros) represent 28.9% of all HIV patients in the French health centers and are particularly mobile, transiting between Paramaribo, French Guiana and Brazil and sometimes seeking medical care in all 3 countries where physicians only know part of their medical and therapeutic history. Finally, it seems most of the burden of HIV concerns Saint Laurent, perhaps because it is a small border city with intense exchanges and more connected sexual networks, but also perhaps because it attracts persons wishing to escape HIV-associated stigma in small villages. Being the largest tertiary care center around, it is often closer than Paramaribo and also presumably attracts patients from the region. The study of data from both MZ and the health centers on the French side, and the recent seroprevalence study along the Maroni suggest that HIV prevalence in the villages along the Maroni is probably lower that in the urban areas, a view that somewhat defies current beliefs but is consistent with network theory which predicts greater transmission in dense urban sexual networks (24–26).

## Human papillomavirus infections

We have recently observed a 33.3% HPV prevalence with a 23.3% prevalence for high risk HPV infections (27). Cervical cancer in French Guiana is the second most frequent cancer among women 24 per 100,000 women with a greater incidence along the Maroni river. Mortality from cervical cancer is 4 per 100,000 women, also higher along the Maroni basin (28). Indeed, 77% of women from rural areas had lesions that had metastasized beyond the cervix, compared to 44% of those from urban areas (29). There are several possible hypotheses for this high incidence of cancer: an early sexual life with multiple partnerships, delays in screening and access to care, immunogenetic differences in terms of susceptibility to HPV viruses, or the circulation of particular oncogenic variants. The Most frequent HPV genotypes were 52, 16, 68, 58, and 31. The analysis of E6 and E7 sequence diversity showed that, for most genotypes, there was a strong ethnic clustering suggesting that Maroons harbored and transmitted viruses that were distinct from Amerindians (30). This possibly reflected different ancestral virus populations and endogamy with rare sexual contacts with other ethnic groups on the Maroni.

Most circulating genotypes are covered by the non-avalent HPV vaccine but, so far, vaccine coverage is lower in French Guiana (14.1%) than in Mainland France (23.7%), and on the Maroni, where HPV is so prevalent, vaccination rates remain close to zero. HPV testing is now authorized and reimbursed in France but beyond research WHO’s 90 × 70 × 90 by 2030 target seems far beyond our reach (90% of girls vaccinated before 15 years old; 70% of women screened by an HPV test twice in their lifetime; 90% of screen-positive women treated). This is the case on both sides of the Maroni river, and should definitely be prioritized if we are to reach the target.

## Hepatitis B

**FIGURE 9:**
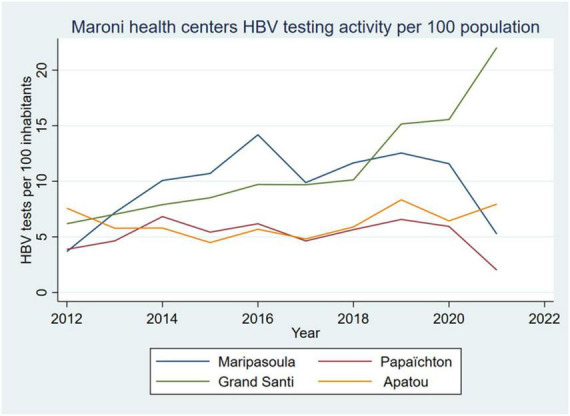
The evolution of the number of HBV tests in the health centers on the French side of the Maroni river show a general increase over time. The disruptions due to COVID-19 have however had an impact in Maripasoula and Papaïchton.

Hepatitis B screening in French Guiana is estimated at 76 per 1,000, the highest figure of all French territories after Guadeloupe (31). Data from the Red Cross STI clinics show a stable prevalence of HbS Ag carriage oscillating around 3% in French Guiana (4% among men and 2% among women). In French Guiana, 37 per 1,000 persons are followed for chronic hepatitis (31). At Saint Laurent du Maroni, in 2021 there were 504 patients with chronic hepatitis B followed at the hospital clinic. Over 56% of patients were aged < 40 years of age; 51.6% were females and 48.4% were males. Among these 504 persons, 13% had a DNA viral load above 20,000 and 13.5% were treated for their virus which is consistent with what is described elsewhere (32). Between 2012 and 2016, the average prevalence of hepatitis B was 2.1%, it was 1.1% in the 4,440 women tested and 3.7% in the 2,902 men tested. In the remote villages of the French side of the Maroni river, a recent cross-sectional study in 666 persons found a 2.1% of positivity for HbS Ag, a prevalence that is the same as that reported in maroons in a previous study (2.1%) but higher than in Amerindians (0.7%) (33). Among gold miners, a cross sectional study found a prevalence of chronic hepatitis B of 4.6% (34). In 2019, before the COVID Epidemic, 79 patients with chronic hepatitis B were seen in the health centers along the Maroni. HBV testing trends have tended to increase (Figure 9) but there was a drop during the COVID-19 epidemic.

The overall average annual testing activity per 100 inhabitants from Apatou to Maripasoula was 8.1 HBV tests per 100 inhabitants, a figure that is greater than in mainland France where it was 6.5 per 100 inhabitants in 2013.

Overall, Hepatitis B prevalence is at an intermediate level, with nearly as many patients with hepatitis B than patients with HIV followed at Saint Laurent du Maroni hospital. The exact vaccination rate is unknown, in 2000, hepatitis B vaccine coverage among children in the interior was low (35); in 2009, HBV vaccination coverage among schoolchildren was suboptimal, at around 75% (31). Since then, a report in 2019 reported that 91% of children had received 3 doses of the hepatitis B vaccine. It thus seems that although younger generations may be well protected, older generations along the Maroni basin may not be, which could explain the significant number of patients with HbS Ag carriage.

In Suriname, the overall HBsAg prevalence in 2012 and 2013 was 2.6%, with significant community disparities [Personal data from Dr. MacDonald-Ottevanger, Dr. Vreden and Dr. Vreden].

## Other sexually transmitted infections

**FIGURE 10:**
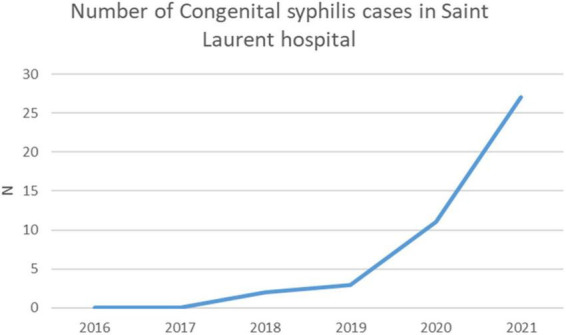
The number of cases of congenital syphilis in Saint Laurent du Maroni has greatly increased during the 2020-2021 COVID-19 pandemic.

The most striking feature in Saint Laurent du Maroni is the recent exponential increase in the number of cases of congenital syphilis (Figure 10), a disease that is targeted for elimination by WHO (36). Global estimates suggest an incidence of congenital syphilis at 473 (385–561) per 100,000 live births (37), which is nearly half of what we observed in 2021, 808 per 100,000 live births (27 cases for 3,340 deliveries). Such an increase has not been observed elsewhere, in Cayenne, in the health centers or the Red Cross. This suggests that the disruptions caused by the COVID 19 pandemic in this border area have hampered normal pregnancy follow-up and that women in that population basin often arrive at the hospital in labor and were never screened for syphilis before, thus paving the way toward congenital infection.

**FIGURE 11:**
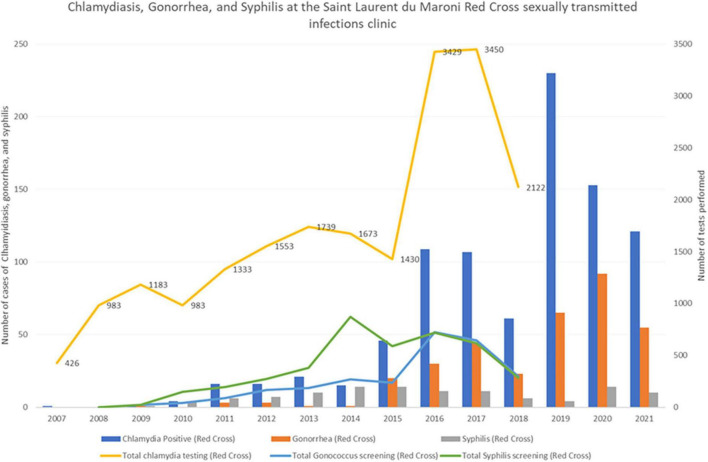
In Saint Laurent du Maroni’s red cross STI clinic testing activity and the number of diagnoses of sexually transmitted diseases (Syphilis, gonorrhea, and chlamydiasis) has greatly increased over time, notably since the addition of PCR.

**FIGURE 12:**
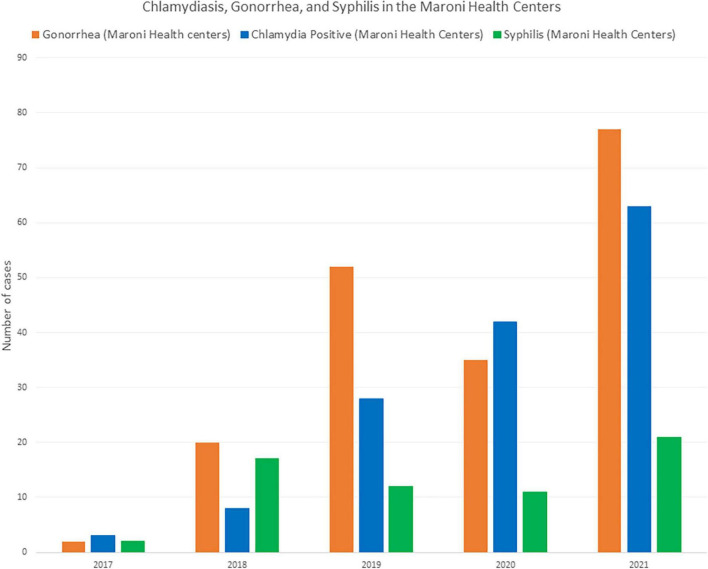
In the health centers on the French side of the Maroni river the number of diagnoses of sexually transmitted diseases (Syphilis, gonorrhea, and chlamydiasis) has greatly increased over time, notably since the addition of PCR.

A recent study in Saint Laurent du Maroni Hospital’s STI clinic found a high incidence of chlamydiasis and gonorrhea (38). Figures 11, 12 show that the incidence of chlamydiasis and Gonorrhea seems to have recently increased both in Saint Laurent and in the health Centers on the French side. However, this may be in part explained by the fact that before 2016, clinicians at the Red Cross mostly relied on a syndromic approach whereas after that the use of PCR allowed to double the number of patients screened and thus diagnose more cases of often asymptomatic sexually transmitted infections. In the health centers the use of PCR started 2 years later and led to a similar increase of the number of diagnoses. In the Maroni health centers on the French side, if we consider a population aged 15-59 of 18312 (39), the 77 cases of gonorrhea in 2021 correspond to an incidence of 4.2 per 1,000 population aged 15-59. For chlamydiasis the 63 cases in 2021 correspond to an incidence of 3.4 per 1,000 population aged 15-59. For syphilis, the incidence was 2.5 per 1,000 population aged 15-59. A master’s thesis in Maripasoula found an incidence of 2.8 per 1,000 women aged 15-59 for Gonorrhea and 6.6 per 1,000 women aged 15-59 for chlamydiasis in 2019 (40). These incidence rates are not particularly high when compared to global estimates in the Americas where chlamydiasis incidence exceeds 60 per 1,000 adult men and women; similarly for gonorrhea the incidence we observed was lower than the overall incidence in the Americas which was estimated at 20 per 1,000 adult men and women (41). These crude comparisons may be criticized as screening efforts and tools may widely differ, as do methods to calculate incidence. Nevertheless, as for HIV prevalence in the remote health centers this low incidence goes against the common belief that gonorrhea and chlamydiasis are widespread in this rural region. The evolution of diagnostic methods and strategies (in 2022 mobile STI clinics should be implemented in Saint Laurent du Maroni) has clearly changed the perception of the problem, but the measurement of the true burden of STIs throughout the Maroni is still a work in progress.

Regarding other sexually transmitted infections (genital Herpes, Trichomonas vaginalis, *Haemophilus ducreyi*), there are no data collected in the health centers or the STI clinic at Saint Laurent Hospital and the red cross. For HTLV-1 –a striking feature of the sexual infection epidemiology on the Maroni— there is no recent data; in 2005, the reported incidence was 0.18 per 100 person-years, seemingly in slight decline, a trend that is likely to have continued given the implementation sustained and deliberate efforts to reduce transmission through breastfeeding. Nevertheless, Acute T Lymphoma/leukemia is the 3rd most common hemopathy in Saint Laurent hospital. HTLV-1 testing is systematically included in all STI screenings.

## Potential drivers of STIs in the Maroni area

Isolation led to a lag of about a decade on the Maroni relative to the coastal cities (16). However, the fact that the HIV epidemic on the Maroni seems to have had an explosive rise in the 1990’s warrants explanations (16). Furthermore, the very high HPV prevalence rate, hepatitis B, HTLV-1 infections (they have however declined overtime due to targeted prevention) also seem to suggest that frequent sexually transmitted infections are a feature of the Maroni basin. A variety of studies have approached the subject. First, some have suggested that the matrilineal (42) organization and the widespread polygamy (43) lead to sexual practices and networks that are conducive to epidemic spread. A recent Ph.D thesis on the Maroni underlined that, still today, both women and men have very different expectations regarding the ideal number of children they wished to have –about 10—when compared to other areas of French Guiana (44). Regarding sexual behaviors, studies documented frequent multiple sexual partnerships in men and recourse to transactional sex. The recent economic crisis, compounded by the COVID 19 shock, are likely to have further increased social and sexual vulnerability, notably transactional sex (45, 46).

Sexual violence was frequently reported with the first intercourse being declared as forced for 6.6% of Maroon women and 12% of Amerindian women (47). Age at first sex was younger than 15 years for 54.4% of maroon men and 33.5% of Amerindian men; for women, 36.5% of maroon women and 49.7% of Amerindian women had their first sexual intercourse before 15 years of age (47).

The Maroni being the “highway” between villages, boatmen, as truckers in Africa, are very mobile and have high risk behaviors (48) thus representing a bridging group between sex workers and different households: hence, 81% declared having multiple sex partners in the past 12 months; 57% used a condom during the last intercourse; 50.4% declared having had sex with sex workers in the past 12 months; and 30% declared at least 1 sexually transmitted infection in the past 12 months (48).

On the French side, since the 1990s, minimum income has been given to persons who were living through a subsistence economy and led to settling and adoption of a monetary economy (49). This well intentioned policy has backfired and fed alcoholism, gambling, and prostitution. However, the upstream portion of the Maroni after Maripasoula is subject to prefectoral authorization to restrict travel and protect Amerindian populations from disease and cultural disruption. This is likely to have had an impact on sexual networks and the diffusion of sexually transmitted infections from downstream toward upstream portions of the Maroni. The interior of French Guiana and Suriname is rich with gold, which attracts thousands of illegal gold miners, mostly from Brazil, and feeds prostitution networks. The prevalence of HIV, syphilis, and hepatitis B has been shown to be important in the gold miner population (50).

Among other potential explanations, cultural practices such as steam baths (51) or boegroes (penile implants making condom use difficult) have been regularly suspected in countries where similar practices occur. Hence, 96.6% of maroon women perform steam baths while only 4.2% of Amerindian women do (47); The hypothesis is that such practices lead to a dry vagina, with a modification of the protective bacterial flora and increased risks for microlesions and subsequent infections. A study in Suriname suggested that regarding *Chlamydia* infection steam baths were not particularly associated with more *Chlamydia* (52). Hypothesizing that, if steam baths made women more vulnerable to sexually transmitted infections (they are usually first practiced after the first menses), there should be a differential incidence for HIV and HTLV-1, we surveyed historical data on both HIV and HTLV-1(53) and there was no such difference which is not in favor of a large effect of vaginal steam baths as a major epidemic driver.

For boegroes, 16.6% of Maroon men and 9.3% of Amerindian men have such penile implants; these implants potentially hamper condom use, favor condom rupture and microlesions in the sex partner. Although these arguments are plausible, we do not have evidence for or against this hypothesis.

Although absence of evidence of an effect is not evidence of absence of effect, it seems that there are already plenty of classical risk factors that can explain the rapid spread of HIV in the 1990s: multiple sex partners, transactional sex, late diagnosis, high populational viral load due to a large proportion of HIV-infected persons with active replication.

## Summary findings and “big picture” of sexually transmitted infections in the region

The Maroni region presents sociocultural, geographical and economic circumstances that have been conducive to the circulation of sexually transmitted infections and to delays in diagnosis and care. So far, Saint Laurent du Maroni seems to be the most affected area, but as a reference center, it is likely that much of the pathology seen there actually comes from elsewhere in the region. Although population groups remain highly endogamous and distance may seem to separate them from the sexual networks along the coast, infectious pathogens eventually reach even the most remote populations. We have seen that HPV infection prevalence reaches levels that are among the highest in the world, with a high cervical cancer incidence and usually more advanced cancers in women living in remote areas. Gonorrhea, *Chlamydia*, hepatitis B, and HTLV-1 are common (38). Great progress has been made to reduce HTLV-1 transmission through breastfeeding (54, 55). Finally, we have seen that since the COVID-19 epidemic, congenital syphilis, a priority for WHO (36), has seen a dramatic increase in Saint Laurent du Maroni and reaches levels that are 40 times higher than in the USA. WHO has emphasized that there is a need for point of care diagnostic tools to combat sexually transmitted infections (56). In French Guiana, both at the STI clinics in Saint Laurent and in the health centers, it has been obvious that the recent introduction of PCR for *Chlamydia* and *Gonococcus* has greatly increased the number of patients screened and the number of diagnoses. In remote centers where mobile patients come and go quickly, results from serologies that require sending blood to Cayenne or Paramaribo will come back too late for action. This emphasizes the importance of rapid tests for hepatitis and syphilis.

## Cooperation

The main limitations of the present scoping review are that we used different anonymous data sources which does not allow to account for population movements, for follow-ups in different hospitals, which may lead to counting the patient both in Suriname and French Guiana. Apart from HIV, the absence of rapid diagnostic tests for other STIs in most of the primary care centers, the reliance on syndromic approaches, and perhaps problems of data reporting leads to underestimate the magnitude of the problem. Nevertheless, this is so far the only effort to shed light on the subject of HIV and sexually transmitted infections on the Suriname-French Guiana border and to conduct a shared analysis by professionals from both sides. Information systems are different, Suriname follows WHO recommendations and indicators while French Guiana follows the French ministry’s format and indicators. The European General Data Protection Regulation (GDPR) applies in French Guiana and complicates data exchange and the principles of open science. The goal of sharing data freely between countries hence seems a bit complicated. However, the present endeavor demonstrates that it is possible to look together at the same problem and bring data from both sides of the Maroni to have a better understanding of it. For that, the very busy and overstretched professionals must take the time to focus on narrow topics of public health importance. Creating and maintaining a cross-border network of health care and public health professionals facilitates the flow of strategic information. Physicians who know each other work with each other easily and may discuss cases when the need arises. Health authorities should thus facilitate such meetings (visas, accommodation, logistics…) to sustain the network even when personnel turnover rate tends to dissolve the nodes and edges of the network.

## A global appraisal of sexually transmitted infections and the need for point of care diagnostic tools

Behavioral change and systematic condom use for casual sex is always a challenge. For hepatitis B and HPV there are available vaccines and while hepatitis B vaccine coverage may be increasing, HPV vaccination –an expensive vaccine—remains exceptional. The curable bacterial STIs may cause severe complications and long-term sequelae. STI case management and secondary prevention by screening or treatment to prevent complications, are greatly hampered by the absence of affordable and accessible diagnostic tests in remote primary health care settings. Case management of STIs mostly rests on syndromic management –genital ulcers, urethral discharge, vaginal discharge, and pelvic inflammatory disease are treated for all the probable causes of these syndromes. This approach has poor specificity, and leads to overprescription of antibiotics, especially in cases of vaginal discharge where most cases are actually caused by vaginosis or candidiasis (57). Perhaps more importantly, the syndromic approach does not disrupt transmission because of the very high proportion of asymptomatic infections. Furthermore, asymptomatic infections may still cause harmful sequelae because of inflammation. Partner notification in these mobile populations remains challenging but some health centers –not all— collaborate with Non-Government Organizations to reach them. Syndromic management dates from an era where diagnostic tests were not available for most STIs and when synergic transmission of HIV and other STIs became recognized (58). However, today, rapid and simple point-of-care tests seem to be promising solutions for more targeted STI case management and control (59, 60). These tests must provide fast turnaround times that allow testing, communication of results, treatment and follow-up plan during the same consultation. The level of advance is not the same for all sexually transmitted infections: for HIV, HBV, Syphilis, there are affordable, highly sensitive, and specific point-of-care tests. However, for chlamydiasis and gonorrhea, the available point-of-care tests have had low accuracy or required expensive equipment. New tests have been recently approved by the FDA (61) but it remains to be seen if they can be implemented and affordable in the context of the Maroni primary care centers. Perhaps, on the French side the implementation of GeneXpert in one health center (accelerated by the COVID 19 epidemic) could allow some progress but again the costs may be a problem (62). The ASSURED benchmark developed by WHO in 2006 is an important guide for the selection of diagnostic tests. Currently, point-of-care tests for syphilis and trichomoniasis meet the ASSURED benchmark but not tests for chlamydiasis or gonorrhea. Although we hope that such tools become available in the setting of the remote villages along the Maroni river, we are aware that there is a great asymmetry in health resources between Suriname and French Guiana. How these tools should be integrated in a binational strategic policy against sexually transmitted infections remains to be determined. This can only be achieved through a coordinated response between the Surinamese and French health professionals.

## Data availability statement

The data analyzed in this study is subject to the following licenses/restrictions: Various datasets were used to shed light on the problem of STIs on the border. Upon reasonable request, some data may be shared after obtaining authorization from the Commission Nationale Informatique et Libertés (CNIL). Requests to access these datasets should be directed to MN, mathieu.nacher@ch-cayenne.fr

## Author contributions

All authors listed have made a substantial, direct, and intellectual contribution to the work, and approved it for publication.
